# Zika virus infection induces mitosis abnormalities and apoptotic cell death of human neural progenitor cells

**DOI:** 10.1038/srep39775

**Published:** 2016-12-23

**Authors:** Bruno S. F. Souza, Gabriela L. A. Sampaio, Ciro S. Pereira, Gubio S. Campos, Silvia I. Sardi, Luiz A. R. Freitas, Claudio P. Figueira, Bruno D. Paredes, Carolina K. V. Nonaka, Carine M. Azevedo, Vinicius P. C. Rocha, Antonio C. Bandeira, Rosalia Mendez-Otero, Ricardo Ribeiro dos Santos, Milena B. P. Soares

**Affiliations:** 1 Gonçalo Moniz Institute, FIOCRUZ, Salvador, Bahia, 40296-710, Brazil; 2 Center for Biotechnology and Cell Therapy, São Rafael Hospital, Salvador, Bahia, 41253-190, Brazil; 3 Laboratory of Virology, Federal University of Bahia, Salvador, Bahia, 40110-100, Brazil; 4 Department of Pathology, Federal University of Bahia Salvador, Bahia, 40110-100, Brazil; 5 Hospital Couto Maia, Salvador, Bahia, 40425-060, Brazil; 6 Institute of Biophysics Carlos Chagas Filho, Federal University of Rio de Janeiro, Rio de Janeiro, Rio de Janeiro, 21941-902, Brazil

## Abstract

Zika virus (ZIKV) infection has been associated with severe complications both in the developing and adult nervous system. To investigate the deleterious effects of ZIKV infection, we used human neural progenitor cells (NPC), derived from induced pluripotent stem cells (iPSC). We found that NPC are highly susceptible to ZIKV and the infection results in cell death. ZIKV infection led to a marked reduction in cell proliferation, ultrastructural alterations and induction of autophagy. Induction of apoptosis of Sox2^+^ cells was demonstrated by activation of caspases 3/7, 8 and 9, and by ultrastructural and flow cytometry analyses. ZIKV-induced death of Sox2^+^ cells was prevented by incubation with the pan-caspase inhibitor, Z-VAD-FMK. By confocal microscopy analysis we found an increased number of cells with supernumerary centrosomes. Live imaging showed a significant increase in mitosis abnormalities, including multipolar spindle, chromosome laggards, micronuclei and death of progeny after cell division. FISH analysis for chromosomes 12 and 17 showed increased frequency of aneuploidy, such as monosomy, trisomy and polyploidy. Our study reinforces the link between ZIKV and abnormalities in the developing human brain, including microcephaly.

Zika virus (ZIKV) is a mosquito-borne Flavivirus first identified in rhesus monkeys in the Zika Forest in Uganda in 1947, and only being reported infecting humans in 1952^1^. After 2007, outbreaks of ZIKV were reported in Micronesia, French Polynesia, New Caledonia, and more recently Latin America^2^,^3^,^4^. After the outbreak of ZIKV in Brazil in 2015, a 20-fold increase in the number of microcephaly cases was observed, establishing a temporal association^5^. The Pan American Health Organization and the World Health Organization issued an epidemiological alert regarding ZIKV infection, congenital malformations and neurological syndromes^6^.

Evidence favoring a causative role for ZIKV in microcephaly has emerged and was the object of several publications. For instance, ZIKV was detected in the amniotic fluids of two fetuses that presented microcephaly, which strongly suggests intrauterine transmission^7^. In addition, detection of the virus together with numerous alterations in the brain of an aborted fetus, while the virus was not detected in any other fetal tissue, also suggested a neurotropism^8^. Epidemiological data showed varied percentage of risk of microcephaly when infection occurs in the first trimester in different geographical locations, suggesting that other factors such as virus strain and co-infections may also contribute to the development of congenital defects^9^. Therefore, the understanding of the mechanisms involved in the neurotoxicity caused by ZIKV is of great relevance.

Studies in animal models have also reinforced the link between ZIKV infection and congenital malformations^10^,^11^,^12^. These, however, do not reproduce properly the human infection, since mice are resistant to ZIKV infection, relying on either type I interferon defective strains, direct injection on fetal cerebral ventricles or injection into the bloodstream of immunocompetent female pregnant mice at extraordinary high titers.

Experimental studies in neural developmental disorders have traditionally been complicated due to the difficulty in obtaining human neuronal cells. Induced pluripotent stem cells (iPSC) were described a decade ago and are a powerful tool for studies of developmental biology and disease modeling^13^. Human iPSCs can be stimulated to undergo neuronal specification and recapitulate several aspects of differentiation and maturation that occur in the normal embryo development. Previous studies using pluripotent-based i*n vitro* neurodevelopment systems have shown that ZIKV infects neural progenitor cells and organoids derived from pluripotent stem cells, impairing cell division^14^,^15^.

Centrosome alterations are closely linked to development of microcephaly, not only due to their role in cell division, but also for their importance in the polarization of neural stem cells^16^,^17^. In the present study, cultures of iPSC-derived cells undergoing neural specification were infected with ZIKV isolated in Brazil during the 2015 outbreak. We show here that ZIKV causes massive death of neural stem cells, which is, at least in part, caused by cell division abnormalities, including the presence of supernumerary centrosomes. Our results reinforce the link between ZIKV infection and the reported defects in central nervous system development.

## Results

### Effects of ZIKV infection in cultures of neural stem and progenitor cells

**Figure 1:**
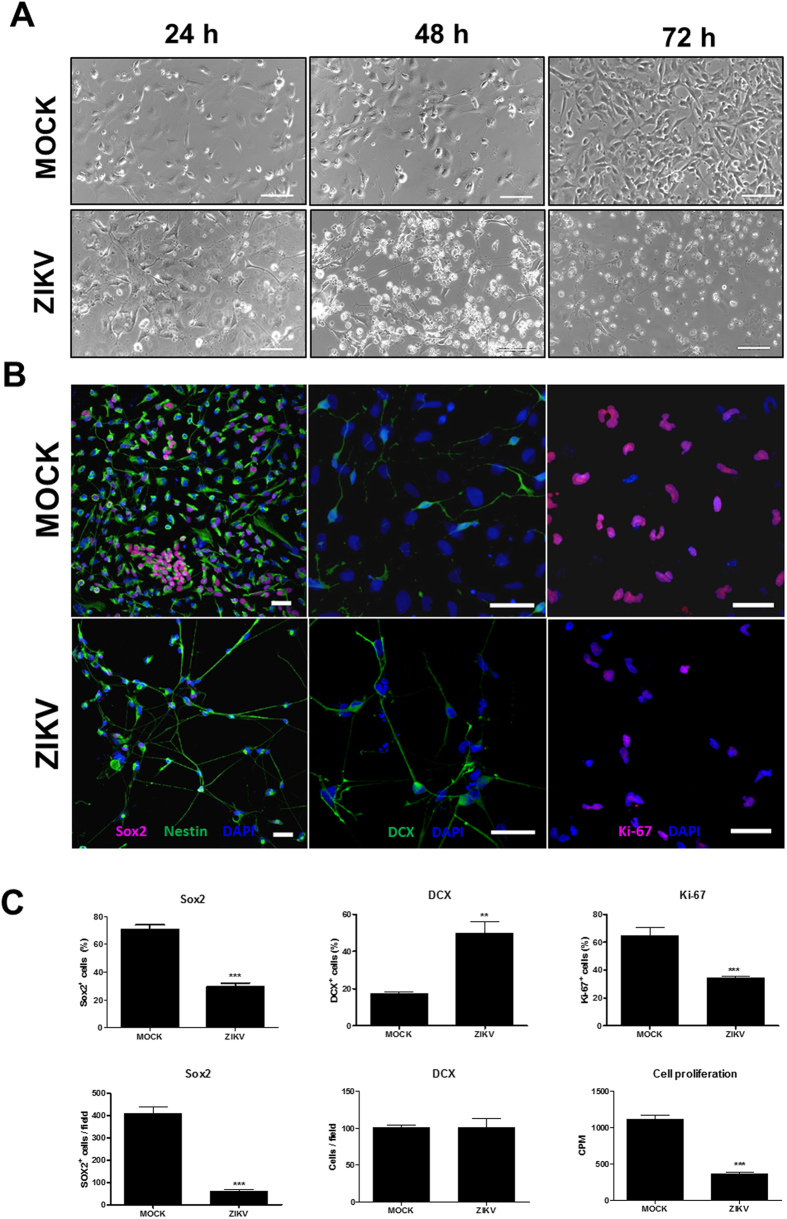
Human iPSC-derived NPC are highly sensitive to ZIKV infection. (**A**) NPC cultures observed by phase contrast 24, 48 and 72 h after ZIKV infection, showing high loss of cells in culture when compared to mock infection. Scale bars = 100 μm. (**B**) Confocal microscopy analyses of mixed NPC cultures immunostained for Sox2, Nestin, DCX, and proliferation marker Ki-67, 72 h after ZIKV or mock infection. Scale bars = 50 μm. (**C**) Quantification of the percentage and density of Sox2^+^ cells, percentage and density of DCX^+^ cells, Ki-67^+^ cells 72 h after mock and ZIKV infection. Cell proliferation was evaluated by ^3^H-thymidine incorporation 48 h after infection. Data represented as mean ± SEM. **p < 0.01; ***p < 0.001.

**Figure 2:**
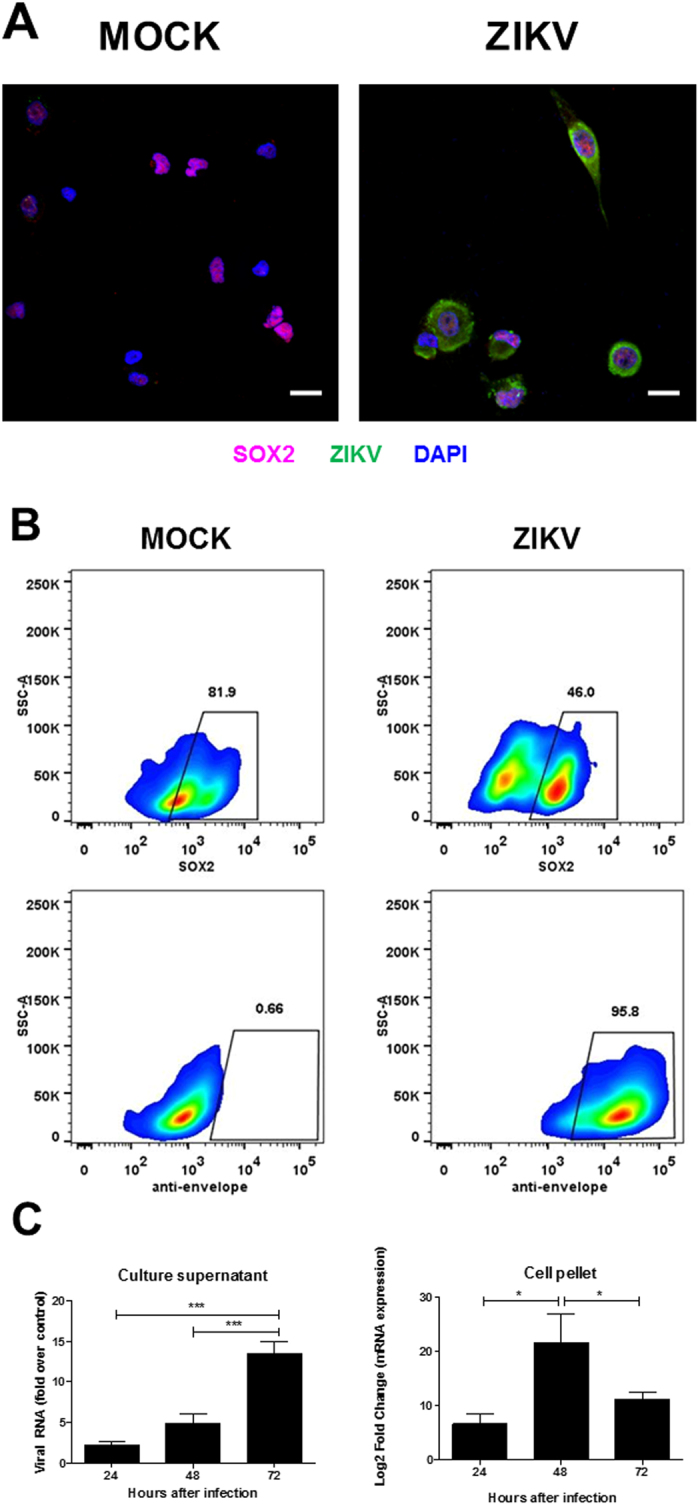
Analysis of ZIKV infection in NPC cultures. (**A**) Immunostaining for ZIKV and Sox2 in NPC cultures. Nuclei are stained with DAPI. Scale bars = 20 μm. (**B**) Flow cytometry analysis for quantification of Sox2^+^ and ZIKV^+^ cells 48 h after infection. (**C**) Quantification of viral RNA in culture supernatants and cell pellets by RT-qPCR 24, 48 and 72 h after ZIKV infection. Data normalized using the values obtained at 2 h time point (supernatant) or endogenous control gene (GAPDH; pellet), and are represented as mean ± SEM. *p < 0.05; ***p < 0.001.

To investigate whether ZIKV infects human NPC, we induced neural differentiation of iPSC obtained by reprogramming human skin fibroblasts (Fig. 1A). The first ZIKV isolate obtained during the outbreak in Brazil was used in the experiments^18^. We performed infections in mixed cell cultures, obtained and expanded from selected neural rosettes after dissociation. At this stage of neural induction, the culture was mainly composed by NPC (70.86 ± 8.3% Nestin^+^ Sox2^+^) and neuroblasts (17.39 ± 1.5% Sox2^−^ DCX^+^). The cells were infected with ZIKV and observed up to 72 h. We observed a marked reduction in cell density with time, in ZIKV-infected cultures when compared with MOCK infected cultures (Fig. 1A). Cytopathic effects of ZIKV were seen as early as 24 h of infection. Although ZIKV infected both Sox2^+^ and Sox2^−^ cells (Fig. 2A), the majority of the remaining cells in ZIKV infected cultures after 72 h of infection were Sox2^−^ (Figs 1B,C and 2B). Mock-infected controls proliferated with time in culture, and most cells expressed Sox2 and Nestin (Fig. 1). Sox2 is a transcription factor expressed in NPC, which is downregulated shortly after neuronal lineage commitment^19^. Therefore, we analyzed the expression of Doublecortin (DCX), a microtubule-associated protein expressed in migrating neuroblasts^20^, in order to evaluate if ZIKV infection would also affect cells at a subsequent stage of neural differentiation. The percentage of DCX^+^ cells in ZIKV-infected NPC cultures was higher than that found in MOCK-infected cultures, although the density of DCX^+^ cells was similar in both groups (Fig. 1B,C). This finding indicates a major susceptibility of NPC to cell death after ZIKV, when compared to neuroblasts. In order to investigate the mechanisms leading to reduction in cell density in NPC cultures, we first evaluated the expression of Ki-67, a proliferation cell marker. The number of Ki-67^+^ cells decreased to approximately 50% in ZIKV-infected NPC, compared to mock-infected controls (Fig. 1B,C). The reduction of proliferation in ZIKV-infected NPC cultures was confirmed using a ^3^H-thymidine incorporation assay (Fig. 1C). Analysis of viral RNA in culture supernatants by RT-qPCR showed increased titers with time of culture, whereas in cell pellets the viral titers increased with 48 h of infection, but reduced at 72 h of infection (Fig. 2C).

### ZIKV induces autophagy and cell death by apoptosis in NPC cultures

**Figure 3:**
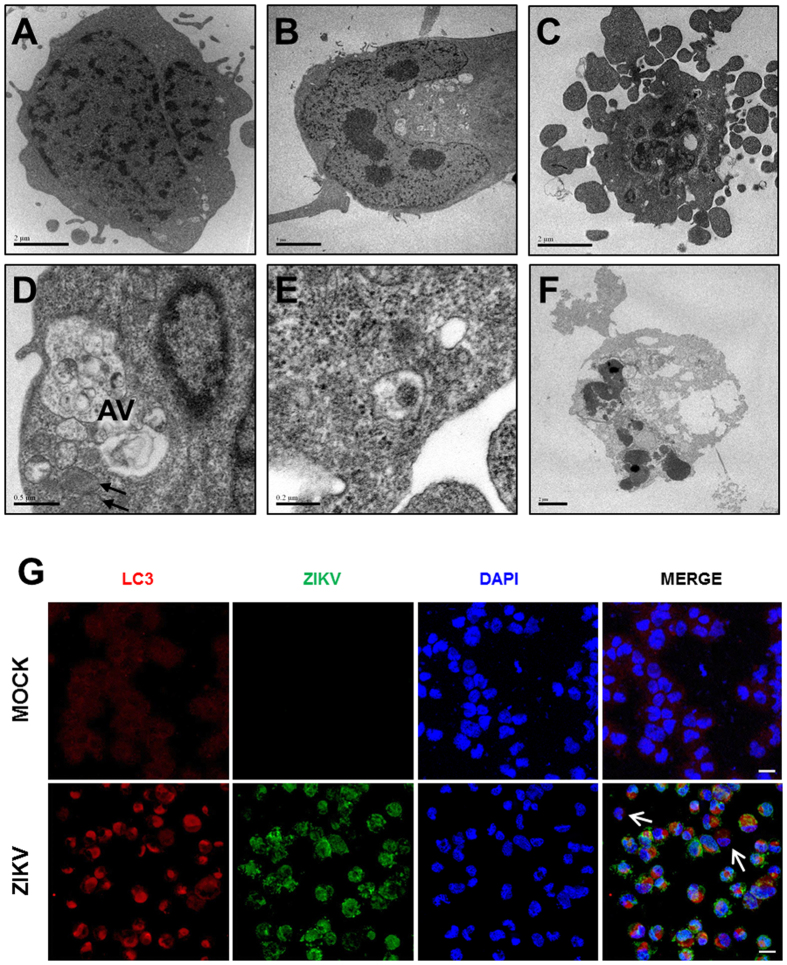
Ultrastructural analysis of NPC cultures after ZIKV infection. (**A**,**B**) Transmission electron micrographs of mock-infected cells, showing nuclei and organelles with normal aspect after 24 and 72 h of culture. (**C–F**) ZIKV-infected cells 24 (**C**,**D**), 48 (**E**) and 72 (**F**) h after infection. Cells in early (**C**) and late (**F**) apoptotic processes. (**D**) The presence of large perinuclear autophagic vacuoles (AV) can be observed. Black arrows indicate mitochondria with altered morphology. (**E**) Presence of viral capsid in intracellular vacuole. (**G**) Immunostaining for ZIKV (green) and the autophagic vacuole marker LC3 (red). Nuclei are stained with DAPI (blue). White arrows indicate cells with negative or low ZIKV staining, and absence of perinuclear LC3 staining. Scale bars = 20 μm.

**Figure 4:**
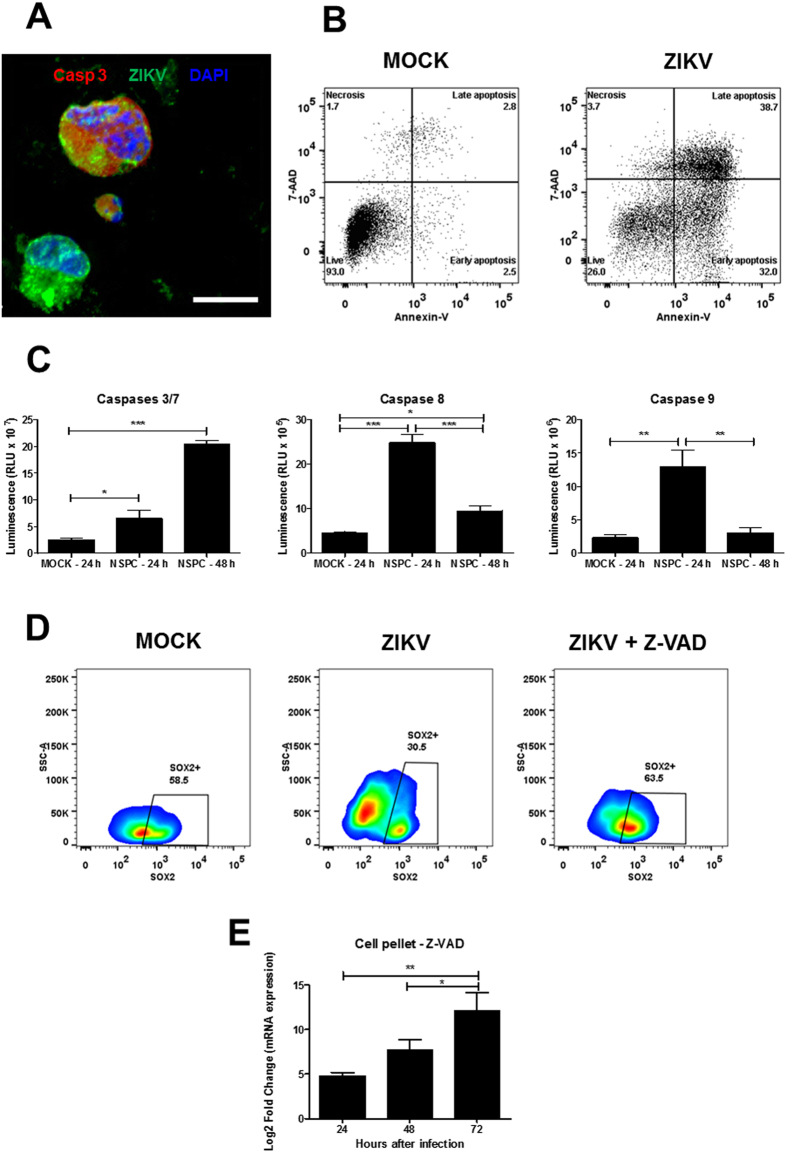
ZIKV infection induces apoptotic cell death in NPC cultures. (**A**) Confocal microscopy of mock and ZIKV-infected NPC stained for cleaved caspase-3 (Casp3; red), DAPI (nuclei; blue), and ZIKV (in green). Scale bars = 20 μm. (**B**) Flow cytometry analysis for detection of apoptosis by Annexin V and 7AAD staining 48 h after mock or ZIKV infection. (**C**) Activity of caspases 3/7, 8 and 9, in mock- and ZIKV-infected cultures was determined using luminescence assays after 24 or 48 h of culture. Data represented as mean ± SEM. *p < 0.05; **p < 0.01; ***p < 0.001. (**D**) Flow cytometry analysis showing the percentage of Sox2^+^ cells in mock-infected and in ZIKV-infected cultures, in the absence and in the presence of Z-VAD (50 μM), 48 h after infection. (**E**) Quantification of viral RNA in cell pellets by RT-qPCR 24, 48 and 72 h after ZIKV infection in the presence of Z-VAD (50 μM). Data were normalized using an endogenous control gene (GAPDH), and are represented as mean ± SEM. *p < 0.05; **p < 0.01.

Ultrastructural analysis by transmission electron microscopy was performed in NPC cultures. In MOCK-infected controls, NPC display a well-preserved morphology, with few small autophagic vacuoles in the cytoplasm and numerous cytoplasmic projections. Nuclei and all organelles displayed a normal aspect (Fig. 3A,B). In contrast, ZIKV infection had a drastic effect on the ultrastructure of NPC. Apoptotic cells were found as early as 24 h after infection, showing condensed fragmented nuclei and numerous autophagic vacuoles in the cytoplasm (Fig. 3C). Numerous and large autophagic vacuoles surrounding the nuclei and altered mitochondria were also observed (Fig. 3D). Viral particles were present inside vesicles (Fig. 3E). After 72 h of infection, the majority of the cells in the field were dead or showing marked cell damage (Fig. 3F). Positive-stranded RNA viruses such as ZIKV induce membrane rearrangements of host cells to replicate their genomes^21^. Autophagy plays physiological roles in recycling proteins and cell organelles, but it can also contribute to cell death if induced in excess^22^. Therefore, we investigated the presence of autophagosomes in ZIKV-infected NPC by confocal microscopy analysis after immunostaining for LC3, a marker of autophagy. We observed positive staining for ZIKV in the majority of the cells 24 h after infection, and this correlated with the presence of a large number of autophagosomes in the perinuclear region. While MOCK-infected control cultures presented a diffuse cytoplasmic pattern and low expression of LC3, ZIKV-infected cells had increased LC3 expression in the perinuclear region (Fig. 3G). Interestingly, cells showing low intensity or absence of ZIKV staining, did not have increased formation autophagic vacuoles, demonstrating a direct effect of the virus in the formation of autophagosomes (Fig. 3G). The mechanisms of cell death in the ZIKV-infected cultures were further investigated by additional markers. The presence of apoptotic NPC in ZIKV-infected cultures was evaluated by confocal microscopy analysis using the expression of cleaved caspase 3 (Casp 3), a central caspase in apoptotic pathways, and nuclear staining with DAPI showing nuclear fragmentation as markers (Fig. 4A). Furthermore, flow cytometry analysis using annexin V and 7AAD staining 48 h after infection also showed increased numbers of NPC in early and late apoptosis in the ZIKV-infected cultures when compared with the MOCK-infected ones (Fig. 4B). Moreover, by luminescence assays, we demonstrated activation of caspases 3/7, as early as 24 h and more intensely after 48 h of infection (Fig. 4C). Caspases 8 and 9 were also significantly activated 24 h after ZIKV infection (Fig. 4C). Finally, incubation of NPC with the pan-caspase inhibitor Z-VAD-FMK prevented the reduction of the population of Sox2^+^ NPC induced by ZIKV 48 h after infection (Fig. 4D). Additionally, Z-VAD-treated cells showed a progressive increase of viral RNA in cell pellets (Fig. 4E).

### ZIKV infection causes mitotic dysfunction in NPC cultures

**Figure 5:**
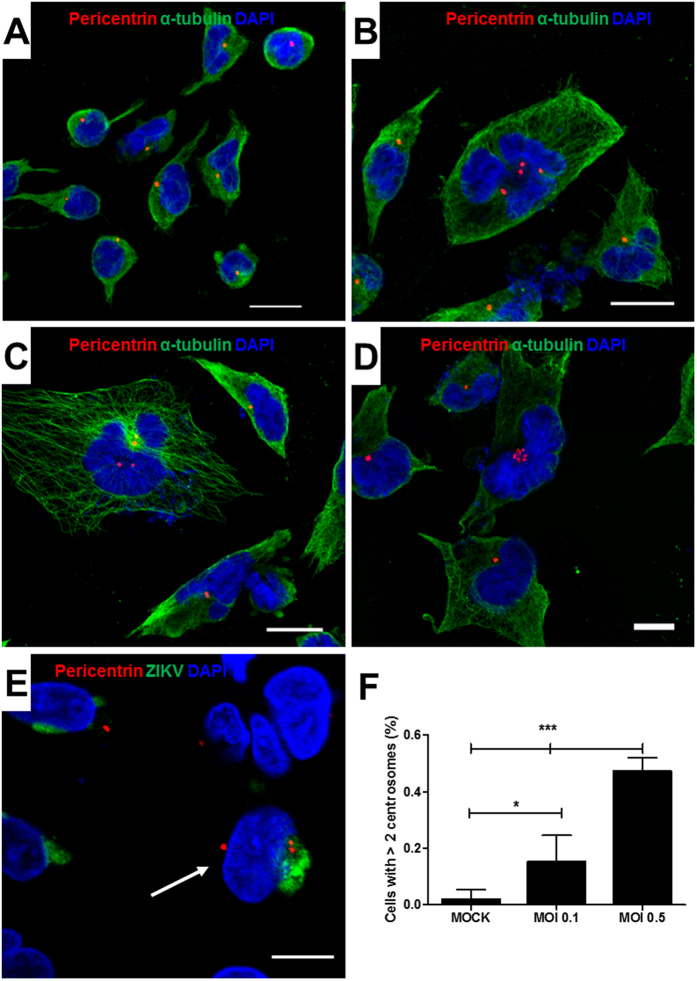
Presence of supernumerary centrosomes in ZIKV-infected cultures. Confocal microscopy of mock (**A**) or ZIKV (**B**–**D**)-infected cells stained for alpha-tubulin (green), DAPI (nuclei; blue), and pericentrin (red), showing cells with more than 2 centrosomes in ZIKV-infected cultures. (**E**) ZIKV-infected cells co-stained with pericentrin (red), ZIKV (green) and DAPI (nuclei; blue), showing the presence of the virus in a cell with three centrosomes (arrow). Scale bars = 20 μm. (**F**) Quantification of cells with supernumerary centrosomes (>2) in mock-infected cultures or in cultures infected with ZIKV at M.O.I. 0.1 and 0.5, 48 h after infection. Data represented as mean ± SEM. *p < 0.05; ***p < 0.001.

**Figure 6:**
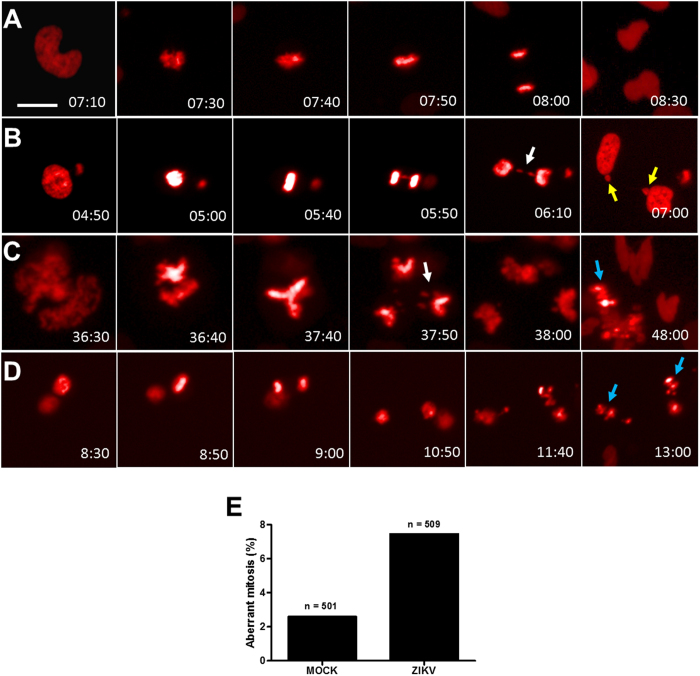
ZIKV infection causes mitotic abnormalities in NPC cultures. (**A**–**D**) Still frames from live imaging experiment showing representative NPC undergoing a bipolar cell division (**A**); bipolar cell division with chromosome lagging and formation of micronuclei (**B**); multipolar cell division with chromosome lagging and loss of one progeny (**C**); and bipolar cell division followed by death in interphase (**D**). Coloured arrows indicate chromosome lagging (white), micronuclei (yellow) and progeny death (blue). (**E**) Percentage of aberrant mitosis in MOCK- and ZIKV-infected NPC cultures. Time, hours:minutes. Scale bars = 20 μm.

**Figure 7:**
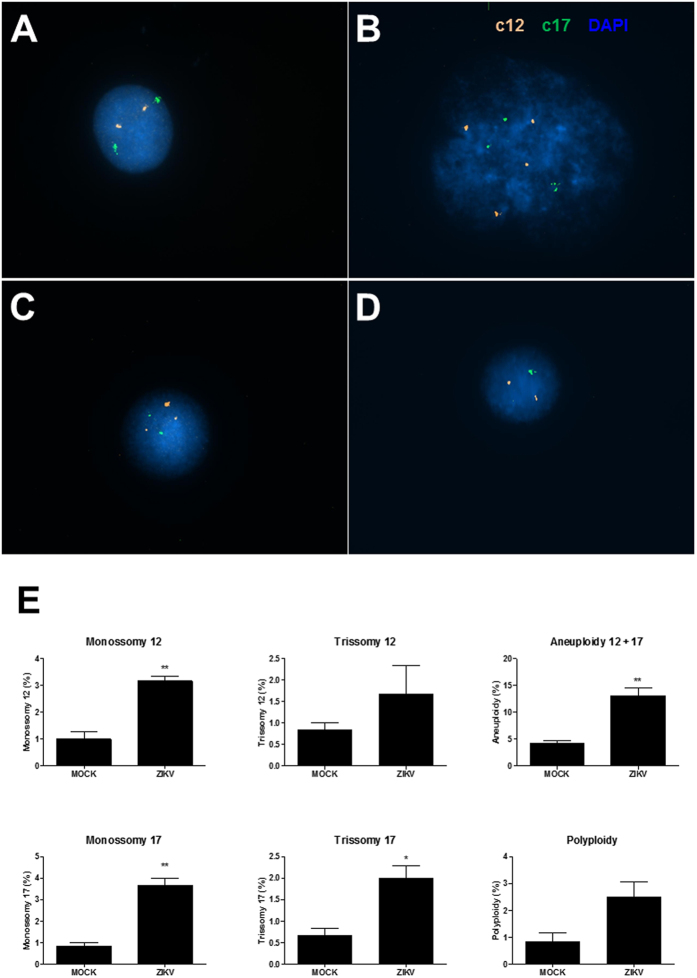
Chromosomal aberrations caused by ZIKV in NPC cultures. Interphase FISH with centromeric probes on mock- (**A**) and ZIKV-infected (**B**–**D**) NPC. (**A**) Euploid cell for chromosome 12 (two orange signals) and chromosome 17 (two green signals). (**B**) Polyploid cell carrying four copies of chromosome 12 and three copies of chromosome 17. (**C**) Trisomy of chromosome 12 (three orange signals). (**D**) Cell carrying a monosomy of chromosome 17. (**E**) Frequencies of numerical chromosomal abnormalities for the chromosomes 12 and 17 analyzed by FISH. The total aneuploidy included the numerical abnormalities (monosomies and trisomies) of chromosomes 12 and 17. Data represented as mean ± SEM. *p < 0.05; **p < 0.01.

Severe mitotic defects have been associated with cell death and microcephaly^17^. To investigate whether ZIKV infection causes cell division abnormalities in NPC, we first analyzed the number of centrosomes in infected cultures. As shown in Fig. 5, ZIKV-infected cultures presented cells with abnormal number (>2) of centrosomes, as shown by pericentrin staining. The presence of ZIKV was seen in cells with extranumerary centrosomes (Fig. 5E). Quantification of cells with extranumerary centrosomes in the ZIKV-infected cultures showed a MOI-dependent dysregulation of centrosome replication (Fig. 5F). To investigate the occurrence of defective mitosis, we generated a genetically modified NPC cell line in which a fusion protein consisting of histone H2b and a reporter gene, mcherry, are expressed, allowing the tracking of genetic material in live cells^23^. The analysis of ZIKV-infected NPC cultures showed the presence of cell divisions with inadequate function of the mitotic apparatus, leading to chromosome lagging and formation of micronuclei (Fig. 6B). Additionally, we found multipolar mitotic spindles, causing loss of progeny (Fig. 6C). Mitotic catastrophe with complete loss of progeny was also observed (Fig. 6D). Quantification by live-imaging of cell divisions showed that abnormal mitoses occur in a higher frequency in ZIKV- infected cultures than in MOCK-infected controls (Fig. 6E). Failure in a proper cell division may lead to the accumulation of aneuploidy. To investigate whether the mitosis defects observed in ZIKV-infected NPC cultures resulted in accumulation of chromosomal abnormalities, we performed FISH analysis for chromosomes 12 and 17. Several chromosome abnormalities, such as polyploidy (Fig. 7B), trisomy (Fig. 7C) and monosomy (Fig. 7D), were detected in ZIKV-infected cultures. Although cells with chromosomal alterations are also found in MOCK-infected cultures, the percentages of alterations were higher after ZIKV infection (Fig. 7).

## Discussion

The results presented herein demonstrate that the Brazilian ZIKV strain is highly lethal to human neural stem/progenitor cells, which express the transcription factor Sox2. This is a critical transcription factor for initiating neural induction and maintaining neural progenitor stem cell properties throughout neural differentiation^19^. A reduction in the population of Sox2^+^ NPC, leading to reduced neuron progeny, has been described previously in a study using iPSC obtained from a patient with primary microcephaly^24^. Our study suggests that ZIKV may cause microcephaly by depleting the founding population of Sox2^+^ NPC, which are highly susceptible to the virus.

Upon *in vitro* infection with ZIKV, NPC show impaired proliferation and rapidly progress to apoptotic cell death, leading to a decrease of viral production at 72 h, which is reversed by treatment with the apoptosis inhibitor Z-VAD. The apoptosis triggered by ZIKV infection in NPC was demonstrated by ultrastructural analysis, flow cytometry and caspase activity assays. Autophagy induction was also observed in NPC cultures after ZIKV, a process that has been previously described for other RNA viruses as a mechanism that favors viral replication^25^. Remarkably, a number of mitotic defects were found in ZIKV-infected cells, including supernumerary centrosomes, multipolarity, incomplete spindle pole separation, formation of micronuclei, and chromosome abnormalities. Some cells entered an apoptotic program in response to the mitotic failure, whereas other cells survived increasing the frequency of aneuploid cells. Thus, because Sox2^+^ cells are on intense proliferation, it is likely that ZIKV infection causes the reduction of cell proliferation and increased apoptosis in part by the induction of mitotic dysfunctions described herein.

Centrosomes are crucial in neurodevelopment, not only for their key role in cell division, but also by participating in cell polarization and migration in the developing brain^17^. One of the immediate consequences of the alteration in the number of centrosomes is the impairment in the chromosomal segregation, which was also found here by evaluating the increasing of aneuploidies. It is also known that aneuploidy itself can be well correlated with death of neural stem cells^17^. We suggest that this could be involved in the origin of brain size reduction in patients infected by Zika virus. Mutations in genes coding for several centrosomal proteins have been associated with the development of microcephaly^16^. The presence of extra centrosomes, as found herein in ZIKV-infected cultures, may disrupt the polarity of the cells *in vivo* and consequently interfere with the development of the brain^16^, resulting in microcephaly and other neurological abnormalities found associated with ZIKV infection. Indeed, neuronal migration defects and lissencephaly have also been described in ZIKV-associated microcephaly cases^26^,^27^. Therefore, the increased numbers of centrosomes and the disruption of chromosomal segregation are important observations, considering that the impairment in these events could imply in consequences even in adult individuals.

A recent report has shown that ZIKV NS4A and NS4B proteins induce the aberrant activation of autophagy in NPC by inhibition of Akt-mTOR signaling, leading to a defective neurogenesis^28^. In our study we also found by immunofluorescence and ultrastructural analysis a massive induction of autophagy in NPC after ZIKV infection. Autophagy induction by Flavivirus has usually been associated with increased viral replication and reduction of cell death^29^. However, since the regulation of autophagic processes is important for cell division due to the high metabolic demand, the aberrant activation of apoptosis induced by ZIKV may contribute to the mitotic failures observed in our study. Moreover, activation of autophagy in adult hippocampal neural stem cells by insulin withdrawal can lead to cell death by inhibition of the Akt-mTOR signaling pathway^22^, reinforcing the importance of autophagy for the biology of NPC.

A previous report using neural progenitor cells derived from human pluripotent stem cells showed attenuated growth of NPC when infected with a Uganda ZIKV isolate^16^. A similar observation using a Brazilian isolate of ZIKV showed the induction of cell death in cultures of human neural stem cells and impairment in the growth of neurospheres and cerebral organoids^17^. In our study we found massive cell death in NPC infected with a Brazilian ZIKV. The comparison of genome sequences of pre-epidemic and epidemic ZIKV strains has indicated possible protein differences, which may be associated with alterations in virulence, tropism or virus replication^30^. Studies aiming to demonstrate putative differences in tropism, neurotoxicity or infectivity of ZIKV isolates are needed.

The marked susceptibility of human Sox2^+^ NPC to ZIKV reinforces the pathological findings in humans, since neural stem cells are present throughout fetal development, postnatal and remain in a few regions of the adult brain^31^. During embryonic development, Sox2^+^ cells are found in the ventricular zone and not only give rise to post-mitotic neurons in the developing brain, but also are need for radial migration of neuroblasts to developing cortex. Our findings reinforce the biological plausibility of the link between ZIKV infection and central nervous system alterations observed in Brazil and French Polynesia, including loss of cortical and cerebellar formation, retinal abnormalities and presence of ventricular calcifications, caused by ZIKV strains sharing a high degree (97–100%) of genetic identity^8^,^26^,^27^,^32^,^33^. Finally, our study indicates the use of iPSC-derived NPC in assays for the development of new intervention methods for the management of ZIKV infection.

## Methods

### Ethics statement

Local ethics committees at São Rafael and Couto Maia Hospitals approved the procedures involving human cells and samples (approval numbers 19883113.0.0000.0048 and 45483115.9.0000.0046, respectively). Written informed consent was obtained from the participants. The experiments were performed in accordance with the approved guidelines.

### ZIKV virus isolation, culture and titration

ZIKV culture was maintained in C6/36. The cells were cultured at 28 °C in Leibovitz L15 medium (ThermoFisher Scientific, Waltham, MA, USA) supplemented with 5% fetal bovine serum (ThermoFisher Scientific), and 10% tryptose phosphate broth (Sigma-Aldrich, St. Louis, MO, USA). C6/36 cells were infected with ZIKV (GenkBank KU940228) isolated from the serum of a ZIKV-infected patient, from Bahia, Brazil. The titration of infectious viruses in the supernatant was performed on VERO cell cultures, obtaining a value of 10^5^ TCID 50/ml.

### NPC induction

Human iPSC generated by reprogramming of skin fibroblasts using episomal vectors^34^. Neural induction was performed by embryoid body formation and incubation in STEMDiff Neural induction Media (Stem Cell Technologies, Vancouver, Canada), according to the manufacturer’s instructions. STEMdiff Neural Rosette Selection Reagent was used for isolation of neural rosettes and NPCs were maintained in StemDiff Neural progenitor media (all from StemCell Technologies, Vancouver, Canada).

### Infections with ZIKV

ZIKV infection was performed in NPC plated on 24-well plates (5 × 10^4^/well) or 6-well plates (2.5 × 10^5^/well) and incubated overnight. On the following day, culture medium was harvested and the cells were incubated with ZIKV diluted for a multiplicity of infection (MOI) of 0.1. Cells were incubated at 37 °C for 1 h and 30 min, when the NPC medium was added to the well. For apoptosis blockage, NPC were pre-incubated with pan-caspase inhibitor 50 μM Z-VAD-FMK (R&D Systems, Minneapolis, MN, USA) for 2 h, followed by ZIKV adsorption step, and incubation for another 46 h.

### Immunofluorescence analyses

The cells were fixed with 4% paraformaldehyde (Electron Microscopy Sciences, Hatfield, PA, USA) for 15 min, permeabilized with 0.1% Triton-X-100 (Sigma-Aldrich) for 15 min and blocked with Protein Block Serum-Free (Dako, Glostrup Municipality, Denmark) for 10 min. For centrosome staining, cells were fixed in ice-cold methanol for 10 minutes, rehydrated in PBS for 5 min, extracted in PBS-0.5% Triton X-100 for 5 min, blocked in TBS-BSA for 30 min, and then incubated with primary antibodies. The following primary antibodies were diluted in 1% BSA/PBS (Sigma-Aldrich) and added to the coverslips: anti-DCX, (1:300; Santa Cruz Biotechnology, Dallas, TX, USA), anti-Nestin (1:200; Millipore, Billerica, MA, USA), anti-Sox2 (1:500; Cell Signaling Technology, Danvers, MA, USA), anti-Ki67 (1:1000; ThermoFisher Scientific), anti-cleaved Caspase-3 (1:400; Cell Signaling Technology), anti-LC3B (1:100; ThermoFisher Scientific); anti-alpha-tubulin (1:1000; Sigma-Aldrich) and anti-pericentrin (1:1000, Abcam, Cambridge, UK). For ZIKV staining, we used a primary monoclonal antibody produced against Flavivirus E protein (MIAF, obtained through the WRECVA, diluted 1:2000), kindly provided by Dr. Nikos Vasilakis (University of Texas, Medical Branch). Alternatively, a serum sample obtained from a local subject with confirmed ZIKV infection in the past 6 months, was used for ZIKV staining, 1:500 dilution (Fig. 6). All the antibodies were incubated overnight at 4 °C. The following secondary antibodies were used, in 1:1000 dilution: anti-mouse IgG Alexa Fluor 488, anti-rabbit IgG Alexa Fluor 568, anti-goat IgG Alexa Fluor 488, anti-mouse IgG Alexa Fluor 488, anti-human IgG Alexa Fluor 488 (all from ThermoFisher Scientific). Nuclei staining was performed with DAPI (Vector Laboratories, Burlingame, CA, USA). Images captured on A1+ confocal microscope (Nikon, Tokyo, Japan) or FluoView 1000 confocal microscope (Olympus, Tokyo, Japan). Quantifications were performed in seven random fields captured under 200x magnification, using the Image Pro Plus v.7.0 software (Media Cybernetics, Rockville, MD, USA.

### Flow cytometry

Cells were incubated in 100 μL of binding buffer (ThermoFisher Scientific) with annexin-V-FITC and 7-AAD (BD Biosciences, San Jose, CA, USA) for 15 minutes in the dark at RT. For intracellular staining, cells were fixed in PFA 1% for 1 h at RT, washed and resuspended with PBS 1X + Triton X-100 0.3% (PBS/Triton), followed by incubation with anti-Sox2 (Santa Cruz Biotechnology) or anti-ZIKV antibodies for 1 h at RT. Cells were washed and incubated for 1 h with anti-goat AlexaFluor 488 or anti-mouse AlexaFluor 488. Samples were then washed and resuspended in PBS for data acquisition with BD FACS DIVA v6.3 on a LSR Fortessa SORP (BD Biosciences) and analysis using FloJo X v10.0.7 (Tree Star, Ashland, OR, USA).

### Cell proliferation assay

NPC were plated in triplicate on 96-well plates (10^4^ cells/well) and cultured at 37 °C in a 5% CO_2_ humidified atmosphere and incubated overnight, followed by infections. One μCi/well of [methyl-^3^H] thymidine (PerkinElmer, Waltham, MA, USA) was added to the cultures 24 h post-infection. Plates were then incubated for 24 h at 37 °C and 5% CO_2_. Incorporation of ^3^H-thymidine was quantified using a β-radiation counter (Chameleon, Hydex; Turku, Finland). Results of cell proliferation were expressed as mean counts per minute (CPM).

### Caspase activity assay

The activity of caspases 3/7, 8 and 9 was measured with Caspase-Glo assay kit, according to the manufacturer’s instructions (Promega, Madison, WI, USA). Briefly, 100 μl of Caspase-Glo reagent was added to each well, mixed with a plate shaker at 300–500 and incubated at room temperature for 2 hours. The luminescence of each sample was measured in a Glomax 20/20 luminometer (Promega).

### Transmission electron microscopy analysis

For transmission electron microscopy, human NPC infected for 24 and 72 h with ZIKV or mock-infected controls were washed with PBS and fixed at 4 °C for 12 h in a solution of 3% glutaraldehyde (Sigma-Aldrich) in PBS. Cells were then washed with 0.1 M sodium cacodylate buffer and post-fixed in osmium tetroxide 1% for 30 min. Dehydration was performed by using a graded series of acetone solutions (from 30–70%) before embedding the samples in epoxy resin Polybed812 (Electron Microscopy Sciences). Ultrathin sections were obtained using EM UC7 ultramicrotome (Leica, Wetzlar, Germany) and contrasted with uranyl acetate and lead citrate. The sections were analyzed using a transmission electron microscope JEM1230 JEOL at 80 Kv.

### Long-term live-cell imaging

NPC lines genetically modified for the expression of H2B-mcherry fusion protein were generated and used in the long-term live-cell imaging experiments. Lentiviral vectors were generated by transient transfection of HEK293FT cells with psPAX2, pMD2G, and PGK-H2BmCherry (Addgene plasmids: #12260, #21217, #12259)^35^. NPCs were transduced with H2B-mCherry lentiviral vectors, expanded and imaged using the Operetta High Content Imaging System (PerkinElmer). The cells were mock- or ZIKV-infected and imaged from 24 to 72 h after infection. The cells were maintained in a chamber with controlled temperature (37 °C) and atmosphere (5% CO_2_). Images were captured at multiple points every 10 min. At least 500 mitoses were analyzed for each condition and were assessed for the presence of chromosome laggards, multipolarity and apoptosis induction during mitosis or interphase.

### Fluorescent *in situ* hybridization (FISH)

The FISH analysis were performed on mock or ZIKV-infected NPC in triplicate. The cells were detached with trypsin/EDTA (ThermoFisher Scientific) and then washed in a hypotonic solution (KCl, 0.075 M). The cells were fixed in Carnoy’s solution (methanol:acetic acid, 3:1) and transferred to slides. The slides were then washed in 2xSSC for 2 min (RT) and in ethanol gradient. Two centromeric probes, one for chromosome 12 (SureFISH Chr12 CEP, Spectrum orange, Agilent Technologies, Santa Clara, CA, USA) and chromosome 17 (SureFISH Chr17 CEP, Spectrum green, Agilent Technologies) were used. The hybridization solution and the samples were denaturated in the slides using Hyper Chrome hybridization chamber (Euroclone, S.p.A., Italy) for 2 minutes at 75 °C. The hybridization step was performed overnight in the Hyper Chrome, at 37 °C. On the next day, the slides were post-washed in 2xSSC/0.3% Igepal for 2 min at 72 °C and in 2xSSC for 5 min at RT. Then, 15 μl the counter stain DAPI:Antifade was applied in the slide, covered with a coverslip and stored in freezer (−20 °C) for 30 min. At least 200 cells were scored per sample by fluorescence microscopy analysis. Only non-overlapping cells with defined bounds were analyzed. Cells carrying more than two signals for one probe were considered trisomic for the correspondent chromosome and cells carrying more than two signals for both chromosomes 12 and 17 were considered polyploid. Cells carrying only one signal for one probe were considered monosomic.

### qRT-PCR analysis

RNA was extracted of cell pellets and culture supernatants using TRIzol^®^ (ThermoFisher Scientific). cDNA was synthetized using High-Capacity cDNA Reverse Transcription Kit (ThermoFisher). The qPCR was prepared with TaqMan^®^ Universal PCR Master Mix (ThermoFisher) and with the oligos previsously described^36^. The relative quantification of both samples was based on the time of viral replication. All reactions were run in triplicates on ABI7500 Fast Real Time PCR (ThermoFisher). The results obtained from cell pellets were normalized to GAPDH, using TaqMan^®^ (ThermoFisher), while the results from culture supernatants were normalized to 2 h post-infection controls. The threshold cycle (2^−ΔΔCt^) method of comparative PCR was used to analyze the results^37^.

### Statistical analysis

All continuous variables are presented as means  ±  SEM. Parametric data were analyzed using unpaired two-tailed *t* tests, for comparisons between two groups, and 1-way ANOVA, followed by Bonferroni post hoc test for multiple-comparison test, using Prism 6.0 (GraphPad Software Inc, La Jolla, CA, USA). Values of *p* < 0.05 were considered statistically significant.

## Additional Information

**How to cite this article**: Souza, B. S. F. *et al*. Zika virus infection induces mitosis abnormalities and apoptotic cell death of human neural progenitor cells. *Sci. Rep.* **6**, 39775; doi: 10.1038/srep39775 (2016).

**Publisher's note:** Springer Nature remains neutral with regard to jurisdictional claims in published maps and institutional affiliations.
